# Current intakes of *trans*-palmitoleic (*trans*-C16:1 n-7) and *trans*-vaccenic (*trans*-C18:1 n-7) acids in France are exclusively ensured by ruminant milk and ruminant meat: A market basket investigation

**DOI:** 10.1016/j.fochx.2020.100081

**Published:** 2020-02-12

**Authors:** Etienne Guillocheau, Clémence Penhoat, Gaëtan Drouin, Ambre Godet, Daniel Catheline, Philippe Legrand, Vincent Rioux

**Affiliations:** a Laboratory of Biochemistry and Human Nutrition, Agrocampus-Ouest, Rennes, France; b French Dairy Interbranch Organization (CNIEL), Technical and Scientific Department, Paris, France

**Keywords:** BMI, body mass index, CHS, cardiovascular health study, DMA, dimethylacetal, FAME, fatty acid methyl ester, GC-FID, gas chromatography-flame ionization detector, GC–MS, gas chromatography-mass spectrometry, HFPS, health professionals follow-up study, MESA, multi-ethnic study of atherosclerosis, NAFLD, non-alcoholic fatty liver disease, NHS, Nurses’ Health Sudy, PHFO, partially hydrogenated fish oil, PHO, partially hydrogenated oil (includes both PHFO and PHVO), PHVO, partially hydrogenated vegetable oil, TLC, thin-layer chromatography, TPA, *trans*-palmitoleic acid, TVA, *trans*-vaccenic acid, Dairy products, Ruminant meat, Ruminant milk, *Trans*-palmitoleic acid, *Trans*-vaccenic acid

## Abstract

High circulating levels of *trans*-palmitoleic acid (TPA) are associated with a lower risk of type 2 diabetes in humans. Thus, the origin of circulating TPA matters. Direct intakes of TPA are ensured by dairy products, and perhaps by partially hydrogenated oils (PHOs). Indirect intakes of TPA rely on dietary *trans*-vaccenic acid (TVA), which occurs in ruminant-derived foods and PHOs. As it is usually assumed that PHOs are not used any longer, we analyzed here a wide range of foods currently available at retail in France. We report that TPA and TVA (1) do occur in ruminant milk and meat, dairy products and in foreign PHOs, (2) do occur in dairy fat-containing foods and (3) do not occur in dairy fat-free foods. Together, our findings demonstrate that ruminant fats are the only contributors to circulating levels of TPA in humans.

## Highlights

• *Trans*-palmitoleic and *trans*-vaccenic acids are fatty acids of nutritional interest.
• Doubts remain about the formal dietary sources of both fatty acids.
• Foods available at retail in France were accurately analyzed.
• Both fatty acids’ intakes are exclusively ensured by ruminant-derived foods.

## 1 Introduction

*trans*-Palmitoleic acid (*trans*-C16:1 n-7 or *trans*-9 C16:1, TPA) was previously identified as a biomarker of metabolic health in several epidemiological prospective studies. High levels of plasma phospholipid TPA were prospectively associated with lower insulin resistance, lower presence of atherogenic dyslipidemia and lower incidence type 2 diabetes in the *Cardiovascular Health Study* (CHS) cohort (Mozaffarian et al., 2010). In the *Multi-Ethnic Study of Atherosclerosis* (MESA) cohort, plasma phospholipid TPA was found to be inversely linked with triglyceridemia, fasting insulin, hypertension and lower risk of type 2 diabetes again (Mozaffarian et al., 2013). In line with these outcomes, Yakoob and colleagues prospectively associated high levels of plasma phospholipid TPA with a lower risk of type 2 diabetes in both the Nurses’ Health Sudy (NHS) and the *Health Professionals Follow-up Study* (HFPS) cohorts (Yakoob et al., 2016). Finally, two *meta*-analyses consistently associated high levels of circulating TPA to lower risk of type 2 diabetes (de Souza et al., 2015, Imamura et al., 2018).

Given these interesting outcomes, unravelling the formal dietary origin of TPA is of importance. Typically, desaturases in humans are unable to synthesize *trans* double bonds, but rather *cis* configurated-double bonds. Thus, one should rely on diet to explain the circulating levels of TPA in humans.

The prevailing view considers direct intakes of TPA that are ensured by ruminant product consumption. First, in the very early 2000s, TPA was indeed reported in ruminant milk available at retail (cow, ewe and goat milk) and accounted for approx. 0.04% of total fatty acids (Destaillats, Wolff, Precht, & Molkentin, 2000). Owing to accurate analytical data, ruminant meat is epidemiologically assumed to contain some TPA (Micha et al., 2010). However, several studies suggest the presence of TPA in partially hydrogenated oils (PHOs) as well. Partial hydrogenation involves of course vegetable oils (PHVOs), and to a lesser extent fish oils (PHFOs). As regards to PHVOs, a significant positive association between their intakes and circulating levels of TPA was found (de Oliveira Otto et al., 2013). Still, the occurrence of TPA in PHVOs has never been formally confirmed by any analytical work to the best of our knowledge. As for PHFOs, accurate analytical work well-demonstrated the presence of TPA among other *trans*-C16:1 positional isomers in these oils (Molkentin & Precht, 1997). Taken together, the data about PHFOs and the assumptions on PHVOs challenge the widespread idea according to which TPA should be considered as a biomarker of dairy fat intake and calls for accurate analysis of PHVOs.

Apart from direct TPA intakes, our research group previously demonstrated that TPA also arises from dietary *trans*-vaccenic acid (*trans*-C18:1 n-7, or *trans*-11 C18:1, TVA) through endogenous peroxisomal β-oxidation (Guillocheau et al., 2019). To explain circulating levels of TPA in humans, one should also look at dietary sources of TVA which are better documented. First, TVA is present in ruminant milk (Wolff, 1995) and ruminant meat (Aldai, Dugan, Rolland, & Kramer, 2009) available at retail and accounts for approx. 2% of the total fatty acids. On the other hand, TVA is among the wide range of *trans*-C18:1 positional isomers generated during partial hydrogenation of fish and vegetable oils (Wolff et al., 2000). Therefore, whatever the content in TPA in PHOs, consumption of such oils indirectly contributes to circulating TPA levels in humans when consumed.

The underlying question is whether PHOs are still used and consumed nowadays, at least in Western countries where regulatory steps against the use of PHOs have been taken. In 2003, the USA and Canada introduced the mandatory labelling of *trans* fatty acid content in foods. Both countries became trailblazers by settling, in 2018, a ban on PHVOs use for human consumption. Concomitantly, there is strong evidence of a decrease in industrial *trans* fatty acid content in foods and consumption in the USA and Canada (Craig-Schmidt & Rong, 2009). As regards to Europe, the European Commission adopted in 2019 a regulatory measure which sets a 2% (% of the total fatty acids) limit for industrial *trans* fatty acids in foods, and that will come into force by April 1st, 2021 (Commission Regulation (EU) 2019/649 of 24 April 2019 Amending Annex III to Regulation (EC) No 1925/2006 of the European Parliament and of the Council as Regards Trans Fat, Other than Trans Fat Naturally Occurring in Fat of Animal Origin, 2019). Meanwhile, several studies clearly pointed out the very low (<1% of total fatty acids) and decreasing levels of industrial *trans* fatty acids in foods available in some European countries. However, a series of market basket investigations also noticed a boundary between Western European countries and Eastern ones, the latter still having *trans* fatty acid contents in popular foodstuffs higher than 5% of total fatty acids (Stender, 2019, Stender et al., 2012, Stender et al., 2014, Stender et al., 2016).

In this article, we aim at assessing the relative contribution of ruminant fats and PHOs to circulating levels of TPA in humans. For this purpose, we assessed the content of TPA and TVA in a wide range of foods available at retail in France. Our hypotheses are the following: (1) TPA and TVA are found in both ruminant fats and PHOs, (2) PHOs are not found any longer in France, making ruminant fats the only contributors to circulating levels of TPA in humans.

## 2 Materials and methods

### 2.1 Food items, lipid extraction and FAME preparation

Ruminant milk, dairy products, ruminant meats, non-ruminant meats and foodstuffs, such as biscuits and ice-creams, were purchased in local supermarkets in Rennes, France between April and June 2018. As regards to ruminant milk and dairy products, not only cow milk-derived foods were included but also ewe and goat milk-derived foods. Likewise, organic dairy products were included even if insights about agricultural practices or species-specificities are beyond the scope of the present work. Concerning biscuits, some were also bought in hard-discount supermarkets at the same location and in the same period of the year. Three vegetable blends that are currently used by food-product corporations in France were obtained from AAK (Malmö, Sweden). Two PHVOs samples were obtained from a European manufacturer (Olenex; Rolle, Switzerland). A PHFO sample was obtained from NOFIMA (Tromsø, Norway): this PHFO used to be employed in former Norwegian margarines available at retail. Products were gathered as follows (see Supplementary Table 1): milk and dairy products (n = 21), ruminant and non-ruminant meat (n = 4), PHOs (n = 3), dairy fat-free foods and blends (n = 14) and dairy fat-containing foods (n = 4).

Lipid extraction from dairy products was realized with hexane/isopropanol (3/2, v/v) as previously performed on the same material (Wolff & Fabien, 1989). Chloroform/methanol (2/1, v/v) was used for lipid extraction from all other foods (*i.e.*, ruminant meat and industrial foods) (Folch, Lees, & Sloane-Stanley, 1957) with slight modifications: the food was homogenized with an Ultra-Turrax® first only with methanol, and second after adding the required volume of chloroform according to Christie’s guidelines (Christie, 1993a).

Fatty acids were afterwards methylated as fatty acid methyl esters (FAME) according to a two-step procedure using first NaOH (0.5 M in methanol) and second BF_3_ (12% in methanol), as previously described (D’Andrea et al., 2002).

As regards to ruminant and non-ruminant meats, dimethylacetals (DMA) and FAME were separated using home-made TLC and silica gel H (ref. 107736; Merck, Darmstadt, Germany). Glass plates were developed with dichloromethane (100%) and sprayed with primuline (5 mg in a 100 ml 80/20 mix of acetone/water; Sigma-Aldrich, Saint-Louis, MO, USA). The spot corresponding to total FAME was scrapped, transferred in a screw-cap tube, and total FAMEs were extracted using methanol/pentane/0.9% NaCl (3/4/3, v/v/v) in this order as previously described (Wolff, 1995). Excess of pentane was removed under of stream of N_2_ at 40 °C.

For each given food, 1 µl of pure total FAME was diluted into 200 µl hexane.

### 2.2 Fractionation of total FAME: analysis of *trans*-C16:1 and *trans*-C18:1 positional isomers

Home-made 0.5 mm-thickness Ag^+^-TLC was performed to fractionate saturated FAMEs, *cis*-FAMEs and *trans*-FAMEs, as previously described (Morris, 1966) using silica gel H (ref. 111695; Merck). Approx. 30 µl of a 300 mg total FAME/ml dichloromethane solution was applied per plate with a syringe (Hamilton, Reno, NV, USA). After development in hexane/ether (90/10) (Wolff, 1995), plates were sprayed with 2,7-dichlorofluorescein (0.02% in ethanol, w/v). The corresponding *trans*-FAME spot eluted between saturated FAMEs and *cis*-FAMEs: this spot was scrapped, transferred in a screw-cap tube and the *trans*-FAME fatty acids were extracted with methanol/pentane/NaCl 0.9% in this accurate order (3/4/3, v/v/v) (Wolff, 1995). Excess of pentane was removed under of stream of N_2_ at 40 °C. The extracted *trans*-FAMEs were finally diluted into 200 µl of hexane.

### 2.3 Total FAME and *trans*-FAME analysis by GC–MS

Total FAMEs were analyzed with an Agilent 7890N gas chromatograph (Agilent Technologies, Santa Clara, CA, USA) equipped with a bonded fused silica capillary column (BPX-70; 60 m × 0.25 mm, 0.25 μm thickness; SGE Analytical Science, Melbourne, Australia). The temperature program started at 150 °C, then increased to 260 °C at 4 °C/min and finally held for 10 min. Helium was used as carrier gas at a constant flow rate of 1.8 ml/min. The split ratio was settled at 5:1, and 0.1 µl of total FAME solution (see Section 2.1.) was injected. Mass spectra were recorded with an Agilent 5975C inert MSD with a triple-axis detector (Agilent Technologies) operated under electron impact ionization conditions (electron energy 70 eV, source temperature 230 °C). Data were obtained in full-scan mode with a mass range of *m*/*z* 50–550 AMU.

*trans*-FAME were analyzed with an Agilent 7890N gas chromatograph (Agilent Technologies) equipped with a bonded fused silica capillary column with higher polarity (BPX-90; 100 m × 0.25 mm, 0.25 μm thickness; SGE Analytical Science). The temperature program started at 120 °C (for 120 min in the case of *trans*-C16:1 analysis; for 240 min in the case of *trans*-C18:1 analysis), then increased to 260 °C at 10 °C/min and held for 10 min. Helium was used as carrier gas at a constant flow rate of 0.8 ml/min. The split ratio was settled at 5:1, and samples were injected with an automatic injector (Agilent Technologies). The injection volume of the *trans*-FAME solution (see Section 2.2.) was either 0.1 µl for *trans*-C16:1 analysis, or 0.5 µl for *trans*-C18:1 analysis. Mass spectra were recorded with an Agilent 5975C MSD (Agilent Technologies). The mass spectrometer was operated under electron impact ionization conditions (electron energy 70 eV, source temperature 230 °C). Data were obtained in full-scan mode with a mass range of *m*/*z* 50–550 AMU.

Identification of positional isomers of both *trans*-C16:1 and *trans*-C18:1 relied on (1) pure standards (Matreya, PA, USA) and (2) *trans*-C16:1 mix kindly provided by Pierluigi Delmonte (FDA, MD, USA), by comparison of retention times. In addition to the retention time, we also used the mass spectra of peaks to verify the correspondence with either a C16:1 fatty acid (*i.e.*, molecular ion M^+^ = 268 AMU) or a C18:1 fatty acid (*i.e.*, molecular ion M^+^ = 296 AMU). Peak integration was performed with MassHunter Workstation Software Qualitative Analysis Version B.07.00 (Agilent Technologies).

The combination of total FAME analysis plus *trans*-C16:1 and *trans*-C18:1 analysis enabled the expression of TPA and TVA as a % of total FAME in a given food (*i.e.*, g/100 g fatty acids). To get an approximative absolute amount of both TPA and TVA in a given product (*i.e.*, g TPA/g product or g TVA/g product), we relied on the labelled lipid content of the food and on the CIQUAL French food composition table (Anses, 2017).

## 3 Results

### 3.1 TPA and TVA do occur in French ruminant milk and dairy products available at retail (n = 21)

**Fig. 1:**
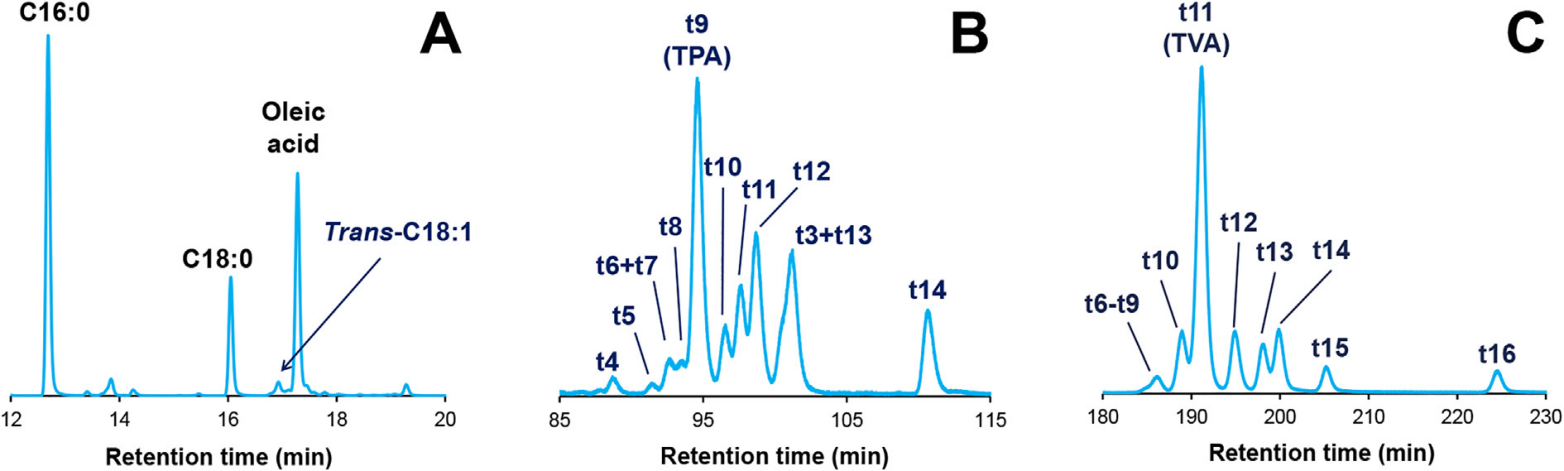
Analysis of *trans*-C16:1 and *trans*-C18:1 FAMEs from a cheese sample currently available at retail in France by GC–MS. (A) Total FAMEs chromatogram: focus on the C18 FAMEs zone. (B) Isomeric distribution of *trans*-C16:1 FAMEs after Ag+-TLC fractionation. (C) Isomeric distribution of *trans*-C18:1 FAMEs after Ag+-TLC fractionation.

Total *trans*-C18:1 but not total *trans*-C16:1 fatty acids were detectable in the total FAME chromatogram of all ruminant dairy foods (see Fig. 1A for a typical total FAME chromatogram of both ruminant milk and dairy products). Mean *trans*-C18:1 content was equal to 2.86% of total fatty acids in dairy foods.

After the Ag^+^-TLC fractionation step, both *trans*-C16:1 and *trans*-C18:1 positional isomers could be resolved for these foods, taking the example of a commercially available cheese (Fig. 1B and Fig. 1C). Among the *trans*-C16:1 isomers, TPA was the major positional isomer. *Trans*-C16:1 having an ethylenic double bond located before the Δ9^th^ position were found in very low amounts. On the contrary, *trans*-C16:1 fatty acids with a double bond located after this Δ9^th^ position are found in higher and different amounts. Of note, relative proportions increase as follows: *trans*-10 < *trans*-11 < *trans*-12. We finally noticed an unusual but already reported overlap between *trans*-3 C16:1 and *trans*-13 C16:1 (Precht & Molkentin, 2000). Concerning the *trans*-C18:1 positional isomers, TVA was found to be the major one by far; other positional isomers were present as well but in lower proportions.

**Fig. 2:**
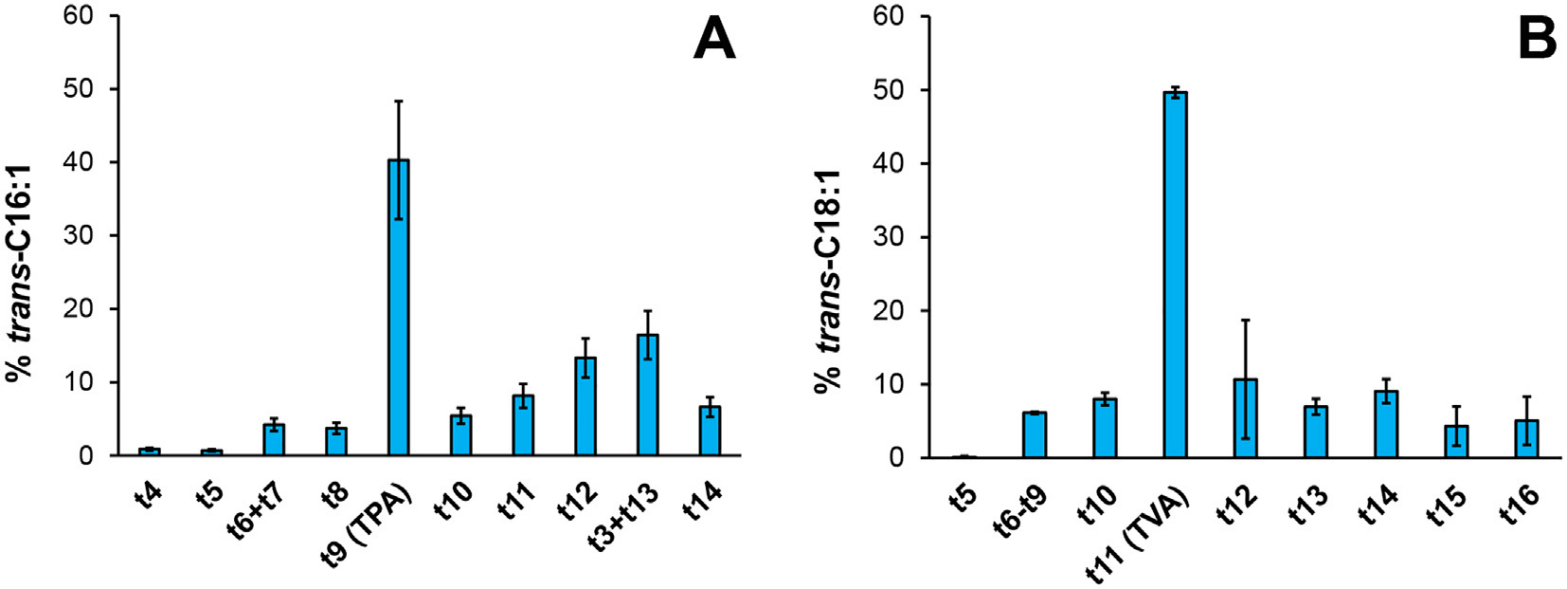
Mean (±SEM, n = 21) distribution of (a) *trans*-C16:1 positional isomers and (b) *trans*-C18:1 positional isomers in ruminant milk and dairy products currently available at retail in France.

Importantly, we found a high consistency as regards to the *trans*-C16:1 and *trans*-C18:1 profiles over the 21 analyzed dairy foods (Fig. 2A and Fig. 2B, respectively). Mean relative content in TPA and TVA was equal to 40.3% of *trans*-C16:1 and 49.6% of total *trans*-C18:1, respectively. Therefore, such a profile can be used as a signature of dairy-foods and helps to characterize both TPA and TVA arising specifically from dairy fat.

Having resolved all positional isomers, we were able to properly assess the contents in TPA and TVA in ruminant milk and dairy products (Table 1). As a mean value established for 21 dairy products, TPA and TVA accounted for 0.030% (±0.004, SEM) and 1.43% (±0.11, SEM), respectively.

**Table 1:** Content in *trans*-palmitoleic acid (*trans*-9 C16:1, or *trans*-C16:1 n-7, TPA) and *trans*-vaccenic acid (*trans*-11 C18:1, or *trans*-C18:1 n-7, TVA) of several ruminant dairy products expressed as both % of total FAME and mg/100 g of a given product.

	*Trans*-palmitoleic acid (TPA)		*Trans*-vaccenic acid TVA	
	% of total FAME	mg/100 g product	% of total FAME	mg/100 g product
Butter (AOP)	0.020	16.3	0.9	750
Butter (regular)	0.017	13.9	0.8	610
*Buttermilk (Lait ribot)*	0.026	0.9	1.4	50
Camembert (organic)	0.081	17	2.6	550
Chavroux®	0.046	6	1.3	170
Cheddar	0.035	6.6	1.5	280
Chocolate dessert cream	0.010	0.3	1.1	30
Concentrated milk	0.015	1.2	2.7	220
Cottage cheese	0.009	0.5	1.3	80
Cream (full-fat)	0.018	2.1	1.1	130
Emmental (organic)	0.041	3.8	1.1	110
Kiri®	0.018	5.3	1.4	400
Milk (cow, full fat)	0.036	1.3	1.2	40
Milk (cow, semi-skimmed)	0.034	0.5	1.3	20
*Petit Suisse*	0.018	0.7	1.5	60
Roquefort	0.020	6.4	1.0	320
Roquefort (organic)	0.060	19.2	1.7	530
Vache Qui Rit®	0.018	3.3	2.0	370
Yoghurt (cow milk, regular)	0.025	0.9	1.6	60
Yoghurt (cow milk, vanilla)	0.031	0.9	1.3	40
Yoghurt (goat milk, regular)	0.059	2.9	1.2	60

### 3.2 TPA and TVA do occur in French ruminant meats available at retail, but not in non-ruminant meats (n = 4)

Total *trans*-C18:1 were detectable in the total FAME chromatogram of the strip-loin steak sample and the lamb sample analyzed, accounting for 4.1 and 4.8% of total fatty acids respectively (Supplementary Fig. 1A and Supplementary Fig. 2A). Like for ruminant milk and dairy products, *trans*-C16:1 were not detectable on the total FAMEs chromatogram.

After the Ag^+^-TLC step, both *trans*-C16:1 and *trans*-C18:1 positional isomers could be resolved for both ruminant meat. As regards to the strip-loin steak, the *trans*-C16:1 profile almost looked like that of dairy products, with TPA being the major positional isomer (Supplementary Fig. 1B). The same holds true for the *trans*-C18:1 profile: TVA is by far the major positional isomer, and other isomers are found in rather low proportions (Supplementary Fig. 1C). TPA and TVA accounted for 0.04% and 2.84% of total fatty acids, respectively.

Concerning the lamb sample, some TPA was found but it was unexpectedly not the major positional isomer (Supplementary Fig. 2B). Likewise, TVA was not the major positional isomer among the *trans*-C18:1 fatty acids in the lamb sample (Supplementary Fig. 2C). In this ruminant meat, TPA and TVA accounted for 0.01% and 0.71% of total fatty acids respectively.

As regards to non-ruminant meat samples, we analyzed a poultry sample and a pork sample. None of these non-ruminant meats displayed any detectable levels of *trans*-C16:1 and *trans*-C18:1 as analyzed by GC–MS (Supplementary Fig. 3A and Supplementary Fig. 3B). More precisely, we were unable to manually integrate on the software what could be considered as total *trans*-C18:1 fatty acids on the total FAMEs chromatogram, due to the closeness with the baseline. Because total *trans*-C18:1 could not be detected in this chromatogram, total *trans*-C16:1 were presumably found at very low levels compared to that in dairy products or in ruminant meats. Therefore, we performed an Ag^+^-TLC fractionation step to specifically analyze the *trans*-FAMEs fraction hoping to resolve at least *trans*-C18:1, but the signal/noise ratio was too low for identifying *trans*-C18:1 isomers.

### 3.3 TPA and TVA are present in currently available foreign partially hydrogenated oils (n = 3)

The analysis of the Norwegian PHFO sample revealed elevated contents not only in *trans*-C18:1, but also in *trans*-C16:1 (Supplementary Fig. 4A). Therefore, the Ag^+^-TLC fractionation step could be easily carried out. A wide range of positional isomers were detected among the *trans*-C16:1 fatty acids, with TPA being the major positional isomer (Supplementary Fig. 4B). Compared with dairy products, *trans*-8 C16:1 was found in a higher relative amount, but *trans*-10 C16:1 is of special concern weighing almost as much as TPA. In fact, a major difference between PHFO and dairy products comes from the positional isomers starting from Δ10^th^ position and that are found in decreasing amounts, *i.e. trans*-10 > *trans*-11 > *trans*-12. This is consistent with a gaussian distribution around the Δ9^th^ position that is encountered after partial hydrogenation. Several positional isomers were also found among the *trans*-C18:1 fatty acids, with *trans*-9 C18:1 being the major positional isomer (Supplementary Fig. 4C). The difference between PHFO and dairy products was even more underlined since *trans*-9 C18:1 was found in lower amounts in the latter.

Unsurprisingly, large amounts of *trans*-C18:1 were noticed in both PHVO samples, that were 28% and 9% of total fatty acids (Supplementary Fig. 5A and Supplementary Fig. 6A). After fractionation of total FAME, we were able to demonstrate the presence of TPA in both PHVOs analyzed (Supplementary Fig. 5B and Supplementary Fig. 6B). Of note, *trans*-C16:1 profiles differed between the two samples: while other *trans*-C16:1 positional isomers and especially *trans*-8 were found in high relative amounts in the first sample, this was not the case for the second in which TPA was undoubtedly the major positional isomer. However, both samples share a common feature that was noticed in the PHFO sample, that is the decreasing relative amounts of *trans*-10 > *trans*-11 > *trans*-12. Again, this result is consistent with the partial hydrogenation process, and clearly makes the difference between TPA coming from dairy fat and TPA from vegetable origin. In both samples, *trans*-C18:1 consisted of a wide range of positional isomers, including TVA (Supplementary Fig. 5C and Supplementary Fig. 6C). From this point of view, a clear difference can be pointed out between TVA coming from a PHVO and TVA arising from dairy fat.

### 3.4 French dairy fat-free foods available at retail contain neither TPA nor TVA, demonstrating that PHOs are no longer used (n = 14)

As demonstrated above, ruminant milk and dairy products do contain TPA and TVA. Because some foods such as biscuits, pastries and ice-creams do contain dairy fat, one should expect to find some TPA and TVA in those. Thus, we first considered biscuits and pastries that do not contain dairy fat to assess the current use of PHOs in France. We also included foods such as currently available different kinds of margarine and hard-discount products.

Like for non-ruminant meat samples, dairy fat-free foods exhibited non-detectable levels of *trans*-C16:1 and *trans*-C18:1 (see Supplementary Fig. 7A). Again, non-detectable levels mean that we were unable to manually integrate a probable total *trans*-C18:1 peak on the total FAMEs chromatogram, due to the closeness to the baseline. An Ag^+^-TLC fractionation was performed, but we failed to identify any *trans*-C16:1 or *trans*-C18:1 positional isomer.

To formally exclude the hypothesis of an occurrence of both TVA and TPA in current vegetable blends, we analyzed three samples of vegetable blends that are used in biscuits and industrial pastries. The total FAME chromatogram showed barely detectable levels of *trans*-C18:1, the highest amount found is 0.02% of total fatty acids in one blend (Supplementary Fig. 7B).

### 3.5 French dairy fat-containing foods available at retail do contain TPA and TVA, because of dairy fat-addition (n = 4)

After analyzing dairy fat-free foods and demonstrating that all were free of TPA and TVA, we focused on dairy fat-containing foods.

The four of the foods considered displayed a total *trans*-C18:1 content between 0.5% and 1.5% of total fatty acids, presumably depending on the relative amount of dairy fat contained in the given food (Supplementary Fig. 8A). After an Ag^+^-TLC step, TPA and TVA were indeed detected in these foods, but the profiles obtained were very similar to that of dairy products and ruminant milk. Regarding the *trans*-C16:1 profile, the increasing amounts of positional isomers with the ethylenic double bond located higher than Δ9^th^, *i.e. trans*-10 < *trans*-11 < *trans*-12, underlined that TPA cannot stem from a PHO, but rather from ruminant fat (Supplementary Fig. 8B). The evidence is clearer concerning the *trans*-C18:1 profile which is mainly characterized by TVA and other positional isomers in a low relative amount (Supplementary Fig. 8C).

## 4 Discussion

In this study, we investigated a wide range of foods to assess the contribution of ruminant milk and meat available at retail to the dietary intake of both TVA and TPA. Because TPA and TVA are natural *trans* fatty acids with a double bond accurately located in the n-7th position of their carbon chain, great attention was paid to the methodology at all stages of the analyses.

As regards to ruminant meat samples, we deemed it important to rely on Folch’s guidelines given that muscle typically contains high amounts of complex phospholipids: should we have relied on a hexane/isopropanol extraction procedure, such lipids might not have been correctly extracted. Acid-catalyzed methylation was chosen since it enables the derivatization of almost all lipid classes contrary to base-catalyzed methylation (Christie, 1993b). However, acid-catalyzed methylation of conjugated linoleic acid was reported to produce methylation artefacts (Kramer et al., 1997). Even if ruminant fat does contain rumenic acid (*cis*-9, *trans*-11 C18:2), we were first interested in TPA and TVA that are not impacted by acid-catalyzed derivatization. We were also aware that acid-catalyzed methylation does generate DMA especially in tissues such as muscle that typically contain high amounts of plasmalogens (Santercole et al., 2007). Since DMA overlaps with *trans*-C16:1 (Guillocheau et al., 2019) and *trans*-C18:1 (Santercole et al., 2007), purification of FAME was carried out to avoid any bias in the assessment of TPA and TVA.

For all foods, fractionation of total FAME was of major importance. This step enables the resolution of *trans* positional isomers (both *trans*-C16:1 and *trans*-C18:1) by working at low temperature (*e.g.*, close to 120 °C) without any overlap with the *cis* counterparts. Unfortunately, a too widespread idea is to consider TPA as the only *trans*-C16:1 isomer whatever the food or the matrix (Destaillats et al., 2000). In fact, TPA assessment by direct GC analysis is usually carried out without appropriate conditions of temperature, so that all *trans*-C16:1 isomers have the same retention time as the TPA authentic standard. Because TPA gets high nutritional coverage, any misunderstanding should be avoided: we strongly recommend that the denomination “total *trans*-C16:1” should be used instead of “TPA/*trans*-9 C16:1/*trans*-C16:1 n-7” whenever the resolution of *trans*-C16:1 positional isomers cannot be assessed. But most important with respect to TPA, the fractionation step removes *iso*-C17:0 contained in ruminant fat. The overlap between TPA and *iso*-C17:0 has already been pointed out as a serious bias with respect to TPA level assessment, not only in the analysis of dairy fat (Destaillats et al., 2000) but also in epidemiological studies (Ratnayake, 2015). Paying attention here to the *iso*-C17:0 makes our data about ruminant fats quite reliable.

Our research group previously analyzed *trans*-C16:1 positional isomers in rat hepatocytes with the BPX-90 column (Guillocheau et al., 2019), but to our knowledge, this is the first food fatty acid compositional analysis carried out with this GC column. Both the AOCS Ce 1 h-05 and the AOCS Ce 1j-07 methods recommend 100 m length CP-Sil 88 and SP-2560 as columns for the accurate analysis of *trans* fatty acids. We also used a 100 m length column with a polarity that we hypothesized to be close to that of CP-Sil 88 and SP-2560. Our chromatograms underline a good resolution of both *trans*-C16:1 isomers and *trans*-C18:1 isomers. Some improvements might be done regarding the *trans*-C16:1 isomers in which the ethylenic bond is located before Δ9, and the *trans*-C18:1 isomers in which the double bond is located before Δ11. For instance, baseline resolution is not achieved in dairy products between (*trans*-6 C16:1 + *trans*-7 C16:1 + *trans*-8 C16:1) and TPA, while previous reports succeeded in getting such a resolution between those peaks (Molkentin & Precht, 2004). The higher film thickness of the BPX-90 (0.25 µm) compared to that of both CP-Sil 88 and SP-2560 (0.20 µm) might be an explanation for this lower resolution.

We found in all ruminant dairy products a highly reproducible profile of both *trans*-C16:1 and *trans*-C18:1 isomers in which TPA and TVA are the major isomers, respectively. Such results are in good agreement with previous studies. It is now well-demonstrated that TVA is the major *trans*-C18:1 isomer in ruminant dairy fat (Wolff, 1995). Our results are also in line with the few studies focusing on *trans*-C16:1 fatty acids in dairy fat and showing that TPA is undoubtedly the major *trans*-C16:1 isomer (Destaillats et al., 2000, Molkentin and Precht, 2004). Of note, we analyzed dairy products derived from different species (*e.g.*, cow, ewe, goat) and from different agriculture management methods (*e.g.*, organic and conventional). In each case interestingly, TPA and TVA remained by far the major *trans*-C16:1 and *trans*-C18:1 positional isomers, respectively. Thus, whatever the dairy product, TPA and TVA are by far the major isomers.

To our knowledge, our study is the first to report the formal presence of TPA in commercially available ruminant meat samples, even if its presence was highly suspected. The occurrence of TVA in ruminant meat is consistent with the ruminal biohydrogenation and in line with previous reports. The role of ruminal biohydrogenation is clearly important towards TPA and TVA since the analysis of non-ruminant meat products showed a very low content in *trans*-C18:1 fatty acids, and presumably so regarding *trans*-C16:1 fatty acids (Belaunzaran et al., 2017). Unfortunately, we were unable to assess the isomeric distribution of *trans*-C18:1 in non-ruminant meat samples due to a too elevated noise after fractionation of FAME. Previous reports also investigated these profiles on non-ruminant meat samples but using Ag^+^solid-phase extraction (SPE) (Belaunzaran et al., 2017). It is possible that self-made Ag^+^-TLC generated higher noise than commercial SPE cartridges. Thus, we believe that our fractionation method can be improved as regards to the analysis of foods containing very low amounts of *trans*-C18:1 fatty acids so that a contribution of non-ruminant meats to circulating levels of TPA in humans cannot be ruled out. In this case, very low levels of TPA and TVA might be explained by the ability of gut microbiota to synthesize TVA from dietary linoleic acid, as demonstrated in rodents (Druart et al., 2013). However, compared to ruminant-derived foods, one can assume that the contribution of non-ruminant meats to circulating levels of TPA is presumably very low.

Higher variability was found in the *trans*-C16:1 and *trans*-C18:1 profiles in ruminant meat samples than in ruminant dairy samples. TPA was the major *trans*-C16:1 isomer in several samples, but not all. Likewise, the major *trans*-C18:1 isomer was TVA, but not for all samples. Similar findings were reported in ruminant meat samples available at retail in Spain (Bravo-Lamas, Barron, Kramer, Etaio, & Aldai, 2016), in the USA (Aldai, Dugan, & Kramer, 2010,), in Canada (Aldai et al., 2009, Aldai et al., 2010) and in Japan (Nagao et al., 2019). Having analyzed only a few ruminant meat samples, we cannot draw any conclusion about how widespread such profiles are in France. More research is therefore needed in this area.

Even if it is certain that TPA arises from ruminal biohydrogenation, the exact pathway is currently not known. Our research group demonstrated that dietary TVA could be chain-shortened to TPA in rodents (Guillocheau et al., 2019), but the same could hold true in ruminants. TVA is a major fatty acid generated during ruminal biohydrogenation, but it is not known if a chain-shortening step takes places in the rumen and/or in the tissues (*e.g.*, mammary gland, muscle). Another hypothesis was made involving the fatty acid C16:3n-3 (*cis*-7,*cis*-10,*cis*-13-C16:3) (Destaillats et al., 2000): assuming that the steps of ruminal biohydrogenation are the same as α-linolenic acid (C18:3n-3, *cis*-9,*cis*-12,*cis*-15 C18:3), TPA can indeed be generated. Further research is needed to know where TPA stems from exactly in ruminants.

To our knowledge, this is the first time that the accurate distribution of *trans*-C16:1 positional isomers from PHVO is reported. Previously, only the amount of total *trans*-C16:1 in PHVOs available in Germany was reported, but the authors could not assess the isomeric distribution due to the low content in *trans*-C16:1 (Molkentin & Precht, 1997). Given the high-variability in content of *trans*-C18:1 in PHVO (Wolff et al., 2000), the same may hold true as well for *trans*-C16:1 in these oils. Thus, we might have analyzed PHVOs with a higher content in *trans*-C16:1 that enabled the assessment of the isomeric distribution. In addition, the previous attempts to analyze *trans*-C16:1 fatty acids in PHVO were carried out with GC-FID (Molkentin & Precht, 1997). In this study, we relied here on GC–MS instead, which is more sensitive and might explain as well why we succeeded in resolving *trans*-C16:1 positional isomers in PHVOs. Finding TPA in PHVOs is in line with what could theoretically happen to *cis*-palmitoleic acid (*cis*-9 C16:1, or *cis* C16:1 n-7) during partial hydrogenation. Our outcomes are also in agreement with epidemiological studies pointing out the role of PHVO in explaining the circulating levels of TPA in humans (de Oliveira Otto et al., 2013).

**Fig. 3:**
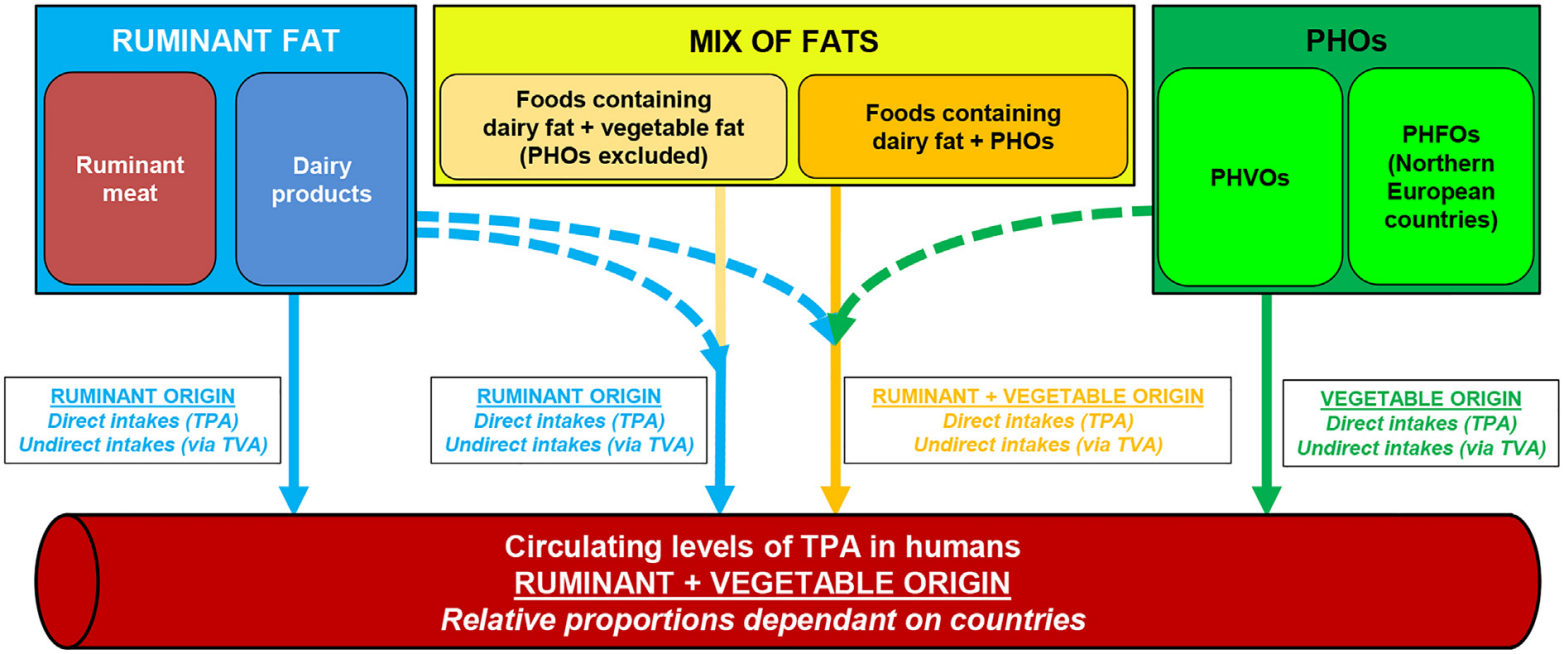
Origin of circulating TPA in humans in the 1990s: contribution of ruminant fats and partially hydrogenated oils to intakes and circulating levels of TPA. *Abbreviations.* PHFO, partially hydrogenated fish oil; PHO, partially hydrogenated oil (includes PHFO and PHVO); PHVO, partially hydrogenated vegetable oil; TPA, *trans*-palmitoleic acid; TVA, *trans*-vaccenic acid.

In almost all epidemiological studies where TPA is associated with metabolic improvements, blood withdrawals of volunteers were realized during the 1990s. At that time, PHVOs were still consumed in high amounts, particularly in the USA where some of these epidemiological studies were carried out. Thus, we hypothesize that in these epidemiological studies the levels of circulating TPA are explained by consumption of ruminant fat and PHVOs: dietary TVA was an already proposed explanation by our research group (Guillocheau et al., 2019), dietary TPA is another one provided by our results (Fig. 3). One can exclude the role of PHFOs at least in the epidemiological studies carried out in the USA since PHFOs were used only in Northern European countries (Mensink & Katan, 1993). Therefore, TPA cannot be considered as a formal biomarker of ruminant fat consumption in the 1990s. The exact relative contribution of dairy fat to TPA intakes (direct plus indirect origin) at that time is however hard to assess. Since ruminant fat accounted for approx. 80% and 50% of dietary TVA intakes in France and in the USA respectively (Wolff et al., 2000), dairy fat should account for up to 80% and 50% of the indirect origin of TPA in these countries at that time. The lack of reliable data about *trans*-C16:1 isomers in foods, plus the high variability found in PHVOs, makes the assessment of ruminant fat contribution to direct TPA intakes in the 1990s difficult. Assuming a closeness between *trans*-C18:1 and *trans*-C16:1 intakes in France, we could reasonably propose a higher contribution of dairy fat than PHVOs to direct TPA intakes at that time.

Despite its past double origin, TPA *itself* might possess some physiological benefits according to epidemiological data. This does not mean that consumption of PHOs is beneficial towards human health; on the contrary, it is now well demonstrated that such consumption is harmful, especially because PHOs also contain a wide range of positional *trans*-C18:1 that are not encountered in dairy fat. Given that high dairy product consumption is significantly associated with a lower risk of type 2 diabetes (Drouin-Chartier et al., 2016), the past double origin of TPA may actually explain why some prospective epidemiological studies carried out in the 1990s failed to report inverse associations between circulating TPA and type 2 diabetes risk, highlighting a neutral impact instead (Kröger et al., 2010, Patel et al., 2010). Therefore, one should focus on the discrepancies between the different cohorts, especially in terms of dietary intakes and popular foods that contributed to circulating TPA.

Considering the current global decrease in the use of PHVOs, we assumed that PHVOs are not used anymore in France and that only ruminant fat currently contributes to TPA and TVA intakes. Our hypothesis was supported by the non-detectable levels of *trans*-C18:1 in dairy fat-free foods, including those bought in hard-discount supermarkets. *Trans*-C18:1 were not detected in vegetable blends either. These very low levels are in strong agreement with that found in similar foods in different Western European countries and are compatible with some residual *trans*-C18:1 fatty acids that arise from the refining process of all crude vegetable oils. In addition, the detector used in our study was MS, which is more sensitive to the widely used FID, and gives strength to our conclusions. Thus, our findings clearly demonstrate that there is no more contribution of PHVOs to dietary intakes of TPA and TVA in France.

**Fig. 4:**
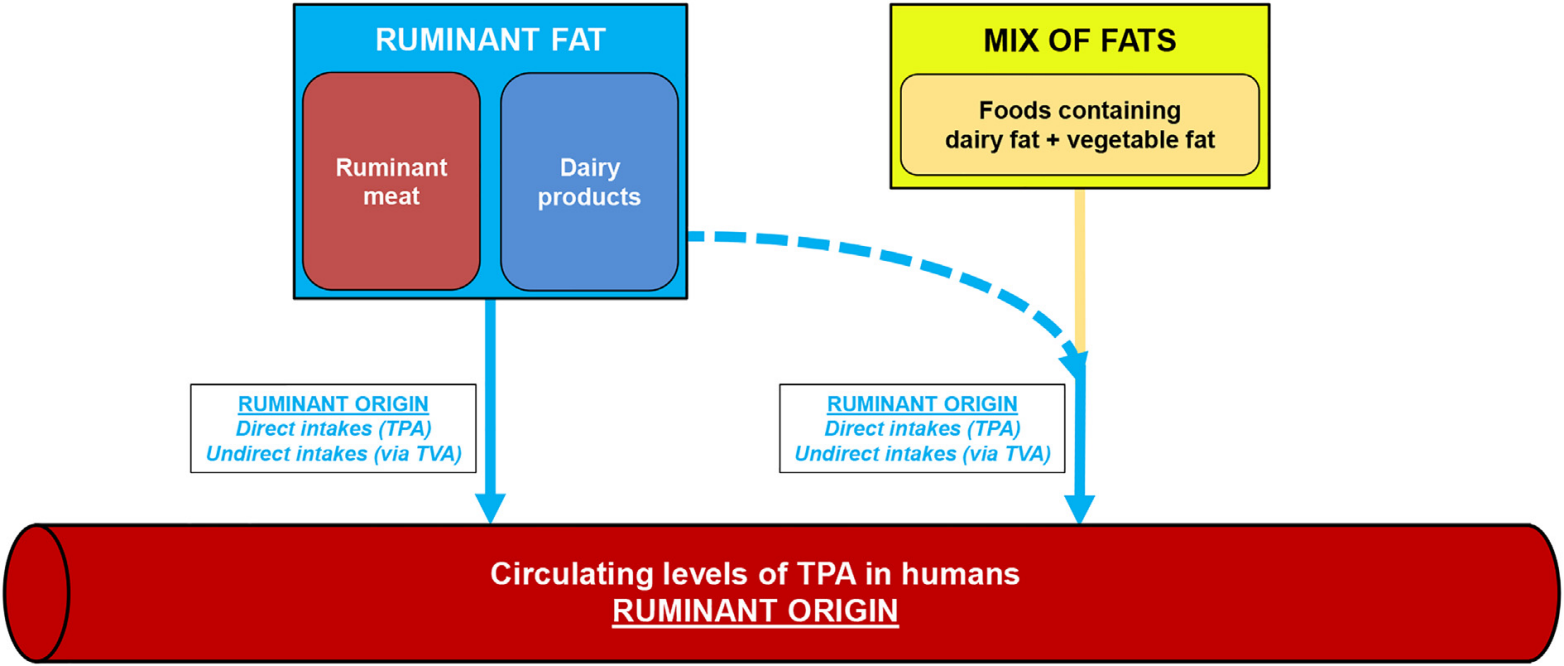
Origin of circulating TPA at the present time and in the forthcoming period: exclusive contribution of ruminant fats to the intakes and circulating levels of TPA in countries where partially hydrogenated oils are not used anymore. *Abbreviations.* PHFO, partially hydrogenated fish oil; PHO, partially hydrogenated oil (includes PHFO and PHVO); PHVO, partially hydrogenated vegetable oil; TPA, *trans*-palmitoleic acid; TVA, *trans*-vaccenic acid.

Further, we formally demonstrated that any TPA and/or TVA detected in food can only arise from dairy-fat addition in this food. This formal demonstration relied on the accurate assessment of both *trans*-C16:1 and *trans*-C18:1 profiles, which enables the distinction between TPA/TVA coming from a PHO and TPA/TVA coming from ruminant fat. Such a demonstration relies on the proper assessment of the *trans*-C16:1 and *trans*-C18:1 profiles. Previous work carried out in Germany in the early 2010s clearly demonstrated that PHOs still contributed to dietary intakes of TVA, relying as well on the *trans*-C18:1 profiles in foods that typically contain a mix of dairy fat and vegetable fat blends (Kuhnt, Baehr, Rohrer, & Jahreis, 2011). Here, we underline that ruminant fats are the only current contributors to circulating levels of TPA in humans in France (Fig. 4). Therefore, further accurate research is needed in Western countries to assess the current use of PHOs in foods.

## 5 Conclusions

In this article, we aimed to assess the relative contribution of ruminant fats and PHOs to circulating levels of TPA in humans. For this purpose, we assessed the content of TPA and TVA in a wide range of foods available at retail in France. We conclude that TPA can be now used as a biomarker of dairy fat intake in the forthcoming period in countries where PHOs are not used any longer.

## Declaration of Competing Interest

The authors declare the following financial interests/personal relationships which may be considered as potential competing interests: EG acknowledges a CIFRE (Industrial Agreement of Training through Research) PhD fellowship from both the French Dairy Interbranch Organization (CNIEL) and the National Association for Research and Technology (ANRT) (CIFRE fellowship number 2015/1195). The present research was financially supported by the French Dairy Interbranch Organization (CNIEL) (PALMITO project) and the Lipids and Nutrition Group (GLN). EG is employed by the French Dairy Interbranch Organization (CNIEL).
