# Supplementary material for: Current intakes of trans-palmitoleic (trans-C16:1 n-7) and trans-vaccenic (trans-C18:1 n-7) acids in France are exclusively ensured by ruminant milk and ruminant meat: A market basket investigation
Source: Food Chem X. 2020 Feb 12;5:100081. doi: 10.1016/j.fochx.2020.100081 (PMC7033321; doi:10.1016/j.fochx.2020.100081)
Supplement: Supplementary data 1 [file mmc1.docx]

**SUPPLEMENTARY TABLE 1**

Detailed list of analyzed products.

| **Type of product** | **Name of product** | **Brand** | **Supermarket/Supplier** | **Origin of lipids** |
| --- | --- | --- | --- | --- |
| **MILK**  **DAIRY PRODUCTS**  **(n = 21)** | Butter (PDO label) | Grand Fermage | Leclerc/Agrial | Animal fat |
|  | Butter (regular) | Paysan-Breton | Leclerc/Laïta | Animal fat |
|  | *Buttermilk (Lait ribot)* | Paysan-Breton | Leclerc/Laïta | Animal fat |
|  | Camembert (organic) | Président | Leclerc/Lactalis | Animal fat |
|  | Chavroux^®^ | Chavroux | Leclerc/Savencia | Animal fat |
|  | Cheddar | Vache Qui Rit | Leclerc/Bel | Animal fat |
|  | Chocolate dessert cream | Danette | Leclerc/Danone | Animal fat |
|  | Concentrated milk | Nestlé | Leclerc/Nestlé | Animal fat |
|  | Cottage cheese (*Faisselle*) | Rians | Leclerc/Triballat | Animal fat |
|  | Cream (full-fat) | Bridélice | Leclerc/Lactalis | Animal fat |
|  | Emmental (organic) | Entremont | Leclerc/Sodiaal | Animal fat |
|  | Kiri^®^ | Kiri | Leclerc/Bel | Animal fat |
|  | Milk (cow, full fat) | Marque-Repère | Leclerc/Leclerc | Animal fat |
|  | Milk (cow, semi-skimmed) | Marque-Repère | Leclerc/Leclerc | Animal fat |
|  | Petit Suisse | Yoplait | Leclerc/Yoplait | Animal fat |
|  | Roquefort | Société | Leclerc/Lactalis | Animal fat |
|  | Roquefort (organic) | Société | Leclerc/Lactalis | Animal fat |
|  | Vache Qui Rit^®^ | Vache Qui Rit | Leclerc/Bel | Animal fat |
|  | Yoghurt (cow milk, regular) | Danone | Leclerc/Danone | Animal fat |
|  | Yoghurt (cow milk, vanilla) | Malo | Leclerc/Sill | Animal fat |
|  | Yoghurt (goat milk, regular) | Rians | Leclerc/Triballat | Animal fat |
| **MEAT**  **(n = 4)** | Beef | Charal | Leclerc/Groupe Bigard | Animal fat (intramuscular only; fat pads removed) |
|  | Lamb | *At retail* | Leclerc/Leclerc | Animal fat (intramuscular only; fat pads removed) |
|  | Pork | *At retail* | Leclerc/Leclerc | Animal fat (intramuscular only; fat pads removed) |
|  | Chicken | Loué | Leclerc/LDC | Animal fat (intramuscular only; fat pads removed) |
| **PARTIALLY HYDROGENATED OILS**  **(n = 3)** | FF 1170 | Olenex | NA^1^/Olenex | Partially hydrogenated rapeseed oil |
|  | CH 4000 | Olenex | NA^1^/Olenex | Partially hydrogenated palm olein |
|  | Partially hydrogenated fish oil | Nofima | NA^1^/Nofima | Partially hydrogenated fish oil^2^ |

^1^Not applicable.

^2^Unspecified oil.

**SUPPLEMENTARY TABLE 1**

*Continued.*

| **Type of product** | **Name of product** | **Brand** | **Supermarket/Supplier** | **Origin of lipids** |
| --- | --- | --- | --- | --- |
| **DAIRY-FAT FREE FOODS**  **(n = 14)** | Ben& Jerry’s Chocolate Fudge Brownie (vegan version) | Ben & Jerry’s | Leclerc/Unilever | Vegetable oils: soybean and coconut oils |
|  | French Fries | McDonald’s | NA^1^/McDonald’s | Vegetable oils^2^ |
|  | Dark chocolate 70% | Lindt | Leclerc/Lindt | Vegetable oil: cocoa butter |
|  | Waffles | Netto | Netto/Netto | Vegetable oils: palm and colza oils |
|  | “Barquette” biscuits | Netto | Netto/Netto | Vegetable oil: colza oil |
|  | BN chocolate | BN | Leclerc/Mondelez | Vegetable oils: palm and colza oils |
|  | Pepito (*pépites*) | LU | Leclerc/Mondelez | Vegetable oil: palm oil |
|  | Oreo (original) | Oreo | Leclerc/Oreo | Vegetable oil: palm oil |
|  | Strawberry roll cake | Netto | Netto/Netto | Vegetable oil: colza oil |
|  | Omega-3 margarine | St Hubert | Netto/Fosun | Vegetable oils: palm; linseed and colza oils |
|  | ProActiv Margarine | Primevère | Leclerc | Vegetable oils: sunflower, palm, linseed and colza oils |
|  | CHOCOFILL™ | AAK | NA^1^ | Vegetable oils: lauric oils |
|  | JASMIN VERT ^TM^ | AAK | NA^1^ | Vegetable oils: palm, coconut and rapeseed oils |
|  | ARKOROMA ^TM^ | AAK | NA^1^ | Vegetable oils: palm oil |
| **DAIRY FAT CONTAINING FOODS**  **(n = 4)** | Ben& Jerry’s Chocolate Fudge Brownie (dairy version) | Ben& Jerry’s | Leclerc/Unilever | Animal fat: cream, concentrated milk  Vegetable oil: soybean oil |
|  | Milk chocolate | Lindt | Leclerc/Lindt | Animal fat: concentrated butter  Vegetable oil: cocoa butter |
|  | Cheese gougeres | Netto | Netto | Animal fat: milk, cheese  Vegetable oils: palm and colza oils |
|  | Schokobons | Kinder | Leclerc/Kinder | Animal fat: milk, concentrated butter  Vegetable oil: cocoa butter |

^1^Not applicable.

^2^Unspecified oil.

**SUPPLEMENTARY FIGURE 1**

Analysis of *trans*-C16:1 and *trans*-C18:1 FAMEs from a strip-loin steak sample currently available at retail in France by GC-MS. **(A)** Total FAMEs chromatogram: focus on the C18 FAMEs zone. **(B)** Isomeric distribution of *trans*-C16:1 FAMEs after Ag+-TLC fractionation. **(C)** Isomeric distribution of *trans*-C18:1 FAMEs after Ag+-TLC fractionation.

| 8  **A** |   **B** |   **C** |
| --- | --- | --- |

**SUPPLEMENTARY FIGURE 2**

Analysis of *trans*-C16:1 and *trans*-C18:1 FAMEs from a lamb sample currently available at retail in France by GC-MS. **(A)** Total FAMEs chromatogram: focus on the C18 FAMEs zone. **(B)** Isomeric distribution of *trans*-C16:1 FAMEs after Ag^+^-TLC fractionation. **(C)** Isomeric distribution of *trans*-C18:1 FAMEs after Ag^+^-TLC fractionation.

|   **A** |   **B** |   **C** |
| --- | --- | --- |

**SUPPLEMENTARY FIGURE 3**

Total FAMEs chromatogram of **(A)** a chicken sample and **(B)** a pork sample currently available at retail in France, analyzed by GC-MS.

|   **A** |   **B** |
| --- | --- |

**SUPPLEMENTARY FIGURE 4**

Analysis of *trans*-C16:1 and *trans*-C18:1 FAMEs from a Norwegian partially hydrogenated fish oil by GC-MS. **(A)** Total FAME chromatogram: focus on the C18 FAMEs zone. **(B)** Isomeric distribution of *trans*-C16:1 FAMEs after Ag^+^-TLC fractionation. **(C)** Isomeric distribution of *trans*-C18:1 FAMEs after Ag^+^-TLC fractionation.

|   **A** |   **B** |   **C** |
| --- | --- | --- |

**SUPPLEMENTARY FIGURE 5**

Analysis of *trans*-C16:1 and *trans*-C18:1 FAMEs from a first European partially hydrogenated vegetable oil by GC-MS. **(A)** Total FAME chromatogram: focus on the C18 FAMEs zone. **(B)** Isomeric distribution of *trans*-C16:1 FAMEs after Ag+-TLC fractionation. **(C)** Isomeric distribution of *trans*-C18:1 FAMEs after Ag+-TLC fractionation.

|   **A** |   **B** |   **C** |
| --- | --- | --- |

**SUPPLEMENTARY FIGURE 6**

Analysis of *trans*-C16:1 and *trans*-C18:1 FAMEs from a second European partially hydrogenated vegetable oil by GC-MS. **(A)** Total FAMEs chromatogram: focus on the C18 FAMEs zone. **(B)** Isomeric distribution of *trans*-C16:1 FAMEs after Ag^+^-TLC fractionation. **(C)** Isomeric distribution of *trans*-C18:1 FAMEs after Ag^+^-TLC fractionation.

|   **A** |   **B** |   **C** |
| --- | --- | --- |

**SUPPLEMENTARY FIGURE 7**

Typical total FAMEs chromatogram of **(A)** dairy fat-free biscuit currently available at retail in France and **(B)** a vegetable fat blend currently used by food-products corporations in France.

|   **A** |   **B** |
| --- | --- |

**SUPPLEMENTARY FIGURE 8**

Analysis of *trans*-C16:1 and *trans*-C18:1 FAMEs from an ice-cream currently available at retail in France, containing dairy fat. **(A)** Total FAME chromatogram: focus on the C18 FAME zone. **(B)** Isomeric distribution of *trans*-C16:1 FAMEs. **(C)** Isomeric distribution of *trans*-C18:1 FAMEs.

|   **A** |   **B** |   **C** |
| --- | --- | --- |
